# Contraceptive use and discontinuation among women in rural North-West Tanzania

**DOI:** 10.1186/s40834-019-0100-6

**Published:** 2019-11-13

**Authors:** Wende Safari, Mark Urassa, Baltazar Mtenga, John Changalucha, James Beard, Kathryn Church, Basia Zaba, Jim Todd

**Affiliations:** 1 National Institute for Medical Research (NIMR), Mwanza Centre, PO Box 1462, Mwanza, Tanzania; 2 London School of Hygiene and Tropical Medicine, London, UK

**Keywords:** Contraception use, HIV status, Life-tables, Retrospective contraceptive calendar

## Abstract

**Introduction:**

Existing estimates of contraceptive use in Tanzania rely on cross-sectional or retrospective study designs. This study used a 2-year, retrospective, month-by-month calendar of contraceptive utilization among women aged 15–49 years.

**Methods:**

We estimated the median duration of contraceptive use, factors associated with use, and contraceptive discontinuation rates in sexually active women, using life tables and Cox proportional hazard model.

**Results:**

A total of 5416 women contributed to the analysis in the study. Of the 5416 women, 942 (17%) had never had sex, 410 (7.6%) had no sexual partner in the last year. Among the 5416 women, 4064 were sexually active during the period, 814 (21.1%) were pregnant or amenorrheic, 610 (15.0%) were using contraception, and 1203 (29.6%) did not want to get pregnant but were not using contraception. In the 1813 women who wanted to avoid pregnancy, contraceptive use was lower among women over 35 years compared to younger ones (OR = 0.28, 95%CI: 0.19, 0.41), and in HIV positive women (OR = 0.89, 95%CI: 0.60–1.32). On the other hand, use was higher among women who were married/living together compared to unmarried ones (OR = 2.23, 95% CI: 1.54, 3.23). Using a 2-year retrospective contraceptive calendar, 1054 women reported contraceptive use, 15.8% discontinued within 6 months and 30.5% discontinued within 12 months. Higher rates of contraceptive discontinuation were observed among women who used pills (OR = 1.86, 95%CI: 1.25, 2.77) or injections (OR = 2.04, 95%CI: 1.59, 2.61) compared to those who used implants.

**Conclusion:**

Contraceptive use was significantly associated with age, education and parity, but not with HIV status. HIV status, number of living children and education are not statistically associated with discontinuation of contraceptive use Pills and injections had the highest rates of discontinuation. Wider choice and greater accessibility of long-acting contraceptive methods with better effectiveness and convenience may serve women better. Furthermore, special efforts may be needed to remove barriers to contraceptive use amongst younger women.

## Introduction

The world population increases by 75 million people each year [1]. Tanzania has an annual fertility rate of 15.4 children per 100 women, with an estimated 1.6 million babies born in 2017 [2]. It is estimated that 20% of pregnancies in Tanzania are unintended or unplanned, with high unmet need for contraception, and limited access to safe abortion. There is a high maternal mortality ratio (MMR) in Tanzania with an estimated 398 [281–570] deaths per 100,000 live births [3, 4]. The challenge remains in Tanzania is to give women the ability to avoid unwanted pregnancy [5].

Despite the efforts that have been made in promoting contraceptive use and family planning in sub-Saharan Africa (SSA), contraceptive practice remains low. The Tanzania Demographic and Health Survey (TDHS) in 2016 showed a significant association between unintended pregnancy and low rates of contraceptive use [5, 6]. The contraceptive prevalence rate amongst women of reproductive age was reported to be 38.4% in 2016 [7]. Of the women who would like to avoid pregnancy, 20% reported they were not using any family planning methods [3, 6].

In some low and middle income countries (LMIC) rates of contraceptive discontinuation remain high and increasing, even among women who want to avoid pregnancy [8]. Results from a 2012 meta-analysis of 60 DHS surveys, which combined all contraceptive methods across 25 LMIC showed that, of women who started using a contraceptive method, on average 38% stopped within the first year, and two thirds (68%) stopped before 2 years [9]. More than half of the women who stopped contraceptive use, had experienced contraceptive failure or had method-related problems, and were still in need of an effective method to prevent unintended pregnancy [9, 10].

Wilcher and others noted that, countries with a high prevalence of HIV also have some of the highest levels of unmet need for family planning (2009) [11]. HIV-positive women have particular needs for contraception to avoid unwanted pregnancy in order to protect their own health, but also to reduce rates of mother-to-child (vertical) transmission of HIV [12]. There is a need to increase access to contraception among women with HIV who do not want to become pregnant, and it is important to integrate family planning with HIV services to meet this need [13]. Strengthening family planning programs will improve the reproductive health outcomes for among women in Tanzania.

The goals of this paper are: first, to provide a more comprehensive picture of contraceptive utilization among sexually active women of reproductive age in rural Tanzania; and second, to estimate contraceptive discontinuation rates from 2013 to 2016 stratifying by HIV status, contraceptive methods, socio-demographic characteristics such as age, marital status, education, and parity.

## Materials and methods

### Study design and population

We have analysed data from cross-sectional epidemiological serological survey (sero-survey) conducted during 2015/2016 nested within the Health and Demographic Surveillance System (HDSS) in Magu district, Tanzania. The Magu HDSS documents births, deaths and migration in a population of over 35,000 in an area of 115 km^2^, as a component of the Kisesa observational HIV cohort study [14]. Every two to 3 years, all residents aged 15 years and above are invited to temporary clinics organized in each of the sero-survey working areas. The sero-survey participants are offered Voluntary Counselling and Testing (VCT), and if found to be HIV- positive are referred to the Care and Treatment Centre (CTC) at Kisesa health centre for treatment.

### Data collection

**Fig. 1:**
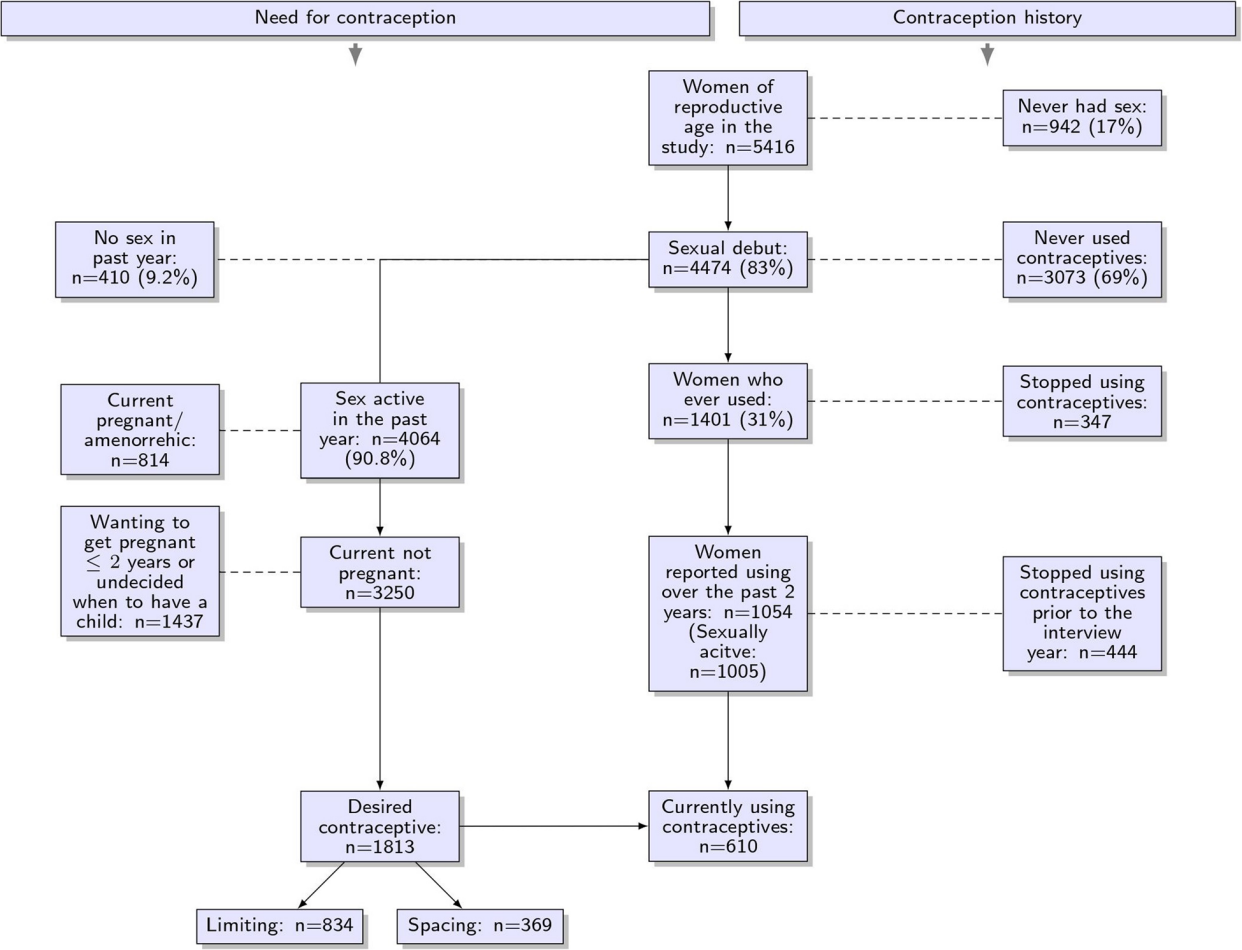
Flowchart of the need for contraception and history of contraceptive use among 5416 women of reproductive age in Kisesa cohort

A structured questionnaire was delivered, by same-sex interviewers using tablet computers, to all HDSS residents attending the sero-survey. All women of reproductive age (15–49 years of age) were asked about their sexual history, whether they had ever used contraceptives, whether they were currently using any contraceptive methods and their history of contraceptive use over the previous 2 years. The consort diagram in Fig. 1 presents the number of women in the study by their sexual history, and contraceptive use.

Data on the two-year history of contraceptive use was collected from all eligible women using a retrospective contraceptive calendar adapted from the 5-year calendar used in DHS surveys [15]. Use of one contraceptive method was recorded separately for each month per woman. From this monthly data, the number of method-specific episodes of contraceptive use were derived. An episode of contraceptive use was defined as the duration in months of continuous use of the same method, that may or may not have ended by the time of interview. For each episode of contraceptive use that ended in discontinuation, a reason for discontinuation was requested. A switch to a new method (or break between methods of at least 1 month) indicated the end of one episode, and the start of a new episode [9].

### Case definition

Women were considered to be sexually active if they reported to have one or more sexual partners in the past year. Contraceptive use was defined as the proportion of all sexually active women who were currently using contraceptives. Women with met needs for contraception were defined as sexually active, non-pregnant women, who were currently using contraception to avoid pregnancy. Women with unmet need for contraception are those who didn’t want to have a child (limiting) or who did not want to have a child in the next 2 years (spacing) but were not currently using contraception (see Fig. 1) [16]. Women were considered HIV negative or HIV positive based on the HIV test at the same sero-survey.

### Statistical analysis

The analysis was restricted to sexually active women, and findings are presented in three sets. Firstly, among all sexually active women a description of the background variables and types of contraceptive use, for both traditional and modern contraceptives.

Secondly, to examine factors associated with current contraceptive use, unadjusted and multivariable logistic regression models were fitted to obtain Odds ratio (OR) with 95% confidence interval (95% CI). This analysis was based on sexually active, non-pregnant women who reported wanting to avoid pregnancy, comparing those using contraceptives and those with unmet need for contraceptives, to assess the effect of each independent variable on the current use of contraceptive methods, and the adjusted effect after controlling for possible confounders.

Finally, from the women who reported contraceptive use during the past 2 years, we constructed a life table of the contraceptive discontinuation rates for the first six and 12 months from the initial use of the contraception. The information was collected in a month-by-month calendar of contraceptive use, from the women who reported use of contraceptives in the past 2 years. The discontinuation rates were calculated overall and for the different contraceptive methods. Cox proportional hazard model was used to obtain adjusted hazard ratios (aHR) and 95% confidence intervals for the association between discontinuation and individual characteristics, the number of living children and the different method types, with discontinuation rates. The unit analysis for this is month of contraceptive use, and the outcome is the discontinuation of contraceptive use [9]. For the analyses of discontinuation rates, the calendar data were truncated at a point 3 months before the interview date to avoid potential bias due to under reporting of first trimester pregnancies [9, 17].

Analyses were done using STATA software, and statistical significance was considered at *p*-value less than 0.05. With 4000 women taking part in the sero-survey, the analysis would have 80% power to detect an 8% difference in contraceptive use between HIV positive and HIV negative women with 5% significance.

### Ethical approval

The eighth round sero-survey had ethical approval from the Lake Zone Institutional Review Board (LZIRB), and the London School of Hygiene & Tropical Medicine (LSHTM). All participants of the eighth sero-survey (including women contributing data for this work) were asked for written informed consent.

## Results

### Background characteristics of the study population

Figure 1 shows the characteristics of 5416 women of reproductive age (15–49 years) in the study population. Among the study respondents, 942 (17%) had never had sex, whereas 4474 (83%) reported sexual debut, and 4064 were sexually active (one or more sexual partner in the last year) and 856 were currently pregnant. Of the 1813 sexually-active, non-pregnant women who did not want another pregnancy at this time, 610 (33.6%) of the women had met need for contraception, and 1203 (66.4%) were not using contraceptives. Of the 1203 women with unmet need in this study, 331 (18.2%) had unmet need for spacing, and 834 (46.0%) had unmet need for limiting.

Excluding 410 (9%) women who were not sexually active, (with no sexual partners in the past year), Table 1 shows the characteristics of the 4064 sexually active women. Eight percent of the sexually active women were HIV-positive and included women aged 15–24 years (34%), 25–34 years (36%), and 35 years and above (30%). Half of women had completed primary school, and only 15% had attended or completed secondary education. More than half of the women (54%) had three or more children.

**Table 1:** Demographic characteristics of 4064 sexually active women in Kisesa from 2015/16

Characteristic	Value (%)
HIV status	
Positive	328 (8.1)
Negative	3724 (91.9)
Age	
15–24	1401 (34.5)
25–34	1474 (36.3)
35+	1189 (29.2)
Education	
None/some primary	1394 (34.3)
Completed primary	2086 (51.4)
Secondary+	580 (14.3)
Number of living children	
*<* 3	1858 (45.7)
3+	2206 (54.3)

### Characteristics of women using contraceptive methods

The contraceptive choices of the 4064 sexually active women (of reproductive age) who had ever had sex is shown in Table 2. In total, 1054 women reported one or more episodes of contraceptive use over the 2 years period prior to the survey, although 49 women were not sexually active in the last year. The most popular contraceptive method was injections with 529 women using this for 535 distinct episodes during the 2 year period (Table 2).

**Table 2:** The background characteristics of 4064 sexually active women in Kisesa in 2015/16 and use of contraceptives over the past two years

Contraceptive history	Pill	Injection	Condom	Implants	IUD	Sterilization	Traditional methods	No. methods	Total no. of women
Ever used	215 (5.3)	812 (20)	22 (0.5)	385 (9.5)	85 (2.1)	17 (0.4)	22 (0.5)	2741 (67.4)	4064
Past 2 years	102 (2.5)	529 (13.1)	14 (0.3)	328 (8.1)	56 (1.4)	15 (0.4)	21 (0.5)	3059 (75.3)	4064
Past 2 years segment used	103	535	17	330	56	15	21		
Background characteristics on contraceptive use in the past 2 years									
HIV status									
Positive	5 (1.5)	39 (11.9)	0 (0)	25 (7.6)	5 (1.5)	4 (1.2)	3 (0.9)	244 (74.4)	328
Negative	97 (2.6)	488 (13.1)	14 (0.4)	302 (8.1)	51 (1.4)	11 (0.3)	18 (0.5)	2811 (75.5)	3724
Age									
15–24	16 (1.1)	127 (9.1)	8 (0.6)	101 (7.2)	9 (0.6)	0 (0)	3 (0.2)	1163 (83.0)	1401
25–34	53 (3.4)	278 (18.9)	3 (0.2)	173 (11.7)	23 (15.6)	2 (0.1)	7 (0.5)	969 (65.7)	1474
35+	33 (2.8)	124 (10.4)	3 (0.3)	54 (4.5)	24 (2.0)	13 (1.1)	11 (0.9)	931 (78.3)	1189
Education									
None/some primary	25 (1.8)	113 (8.1)	3 (0.2)	90 (6.5)	16 (1.1)	7 (0.5)	4 (0.3)	1141 (81.9)	1394
Completed primary	62 (3.0)	308 (14.8)	4 (0.2)	176 (8.4)	31 (1.5)	8 (0.5)	10 (0.5)	1490 (71.4)	2086
Secondary+	15 (2.6)	62 (10.7)	7 (1.2)	62 (10.7)	9 (1.6)	0 (0)	7 (1.2)	429 (74.0)	580
Number of living children									
*<* 3	38 (2.0)	159 (8.6)	10 (0.5)	121 (6.5)	14 (0.8)	0 (0.0)	4 (0.2)	1531 (82.4)	1858
3+	64 (2.9)	319 (14.5)	4 (0.2)	207 (9.4)	42 (1.9)	15 (0.7)	17 (0.8)	1546 (70.1)	2206

As shown in Table 2, injections and implants were chosen more often by HIV positive women. Injectables were chosen more often by women who have completed primary school, users who are 25–34 years old and users with three or more children. Implants were chosen more often by women with post primary education.

### Factors associated with current contraceptive use

Table 3 presents unadjusted and adjusted effects of the predictors for currently using contraceptives among the 1813 sexually active, non-pregnant women wanting to avoid pregnancy. There was an independent effect of age, education and marital status on the current use of contraceptives by these women (Table 3). Compared to the youngest age group (15–24 years), the odds of currently using contraceptive are 0.28 times for women who are 35 years and above (OR = 0.28, 95% CI: 0.19, 0.41). Contraceptive use was lower among HIV positive women compared to HIV negative women although this effect was not significant (OR = 0.89, 95%CI: 0.60–1.32). On the other hand, the odds of current use of contraceptives are higher among women who completed primary education or those with post primary school (OR = 1.67, 95% CI: 1.32, 2.13; OR = 1.95, 95% CI: 1.36, 2.80, respectively) compared to women with no formal education. Notably, contraceptives use varied by marital status and was significantly higher among women who were married/ cohabiting (OR; 2.23, 95% CI 1.54, 3.23) and those who were divorced/separated/widowed (OR; 2.14, 95% CI 1.33, 3.44) as compared to unmarried ones.

**Table 3:** The odds ratio for the determinants of current use of contraception among 1813 non-pregnant, sexually active women of reproductive age, who say they do not want to get pregnant

			Unadjusted model		Adjusted model	
Categories	Total	Current use	Odds Ratio		Odds Ratio	
	*N* = 1813	*N* = 610	(95% C.I.)	*p*-value	(95% C.I.)	*p*-value
Intercept					1.00	
HIV status^a^						
Negative	1651	566	1.00			
Positive	155	42	0.71 (0.49,1.03)	0.0714	0.89 (0.60,1.32)	0.5598
Age						
15–24	373	144	1.00			
25–34	643	312	1.50 (1.16,1.94)	0.0022	1.17 (0.84,1.64)	0.3578
35 and above	797	154	0.38 (0.29,0.50)	<.0001	0.28 (0.19,0.41)	< 0.0001
Martial status						
Unmarried	207	55	1.00			
Married/Living together	1418	494	1.48 (1.07,2.05)	0.0194	2.23 (1.54,3.23)	< 0.0001
Divorced/Separated/Widowed	188	61	1.33 (0.86,2.05)	0.2008	2.14 (1.33,3.44)	0.0017
Education						
None/some primary	593	150	1.00			
Completed primary	977	360	1.72 (1.46,2.18)	< .0001	1.67 (1.32,2.13)	< .0001
Secondary+	270	100	2.11 (1.54,2.89)	< .0001	1.95 (1.36,2.80)	0.0003
Number of living children						
< 3	539	203	1.00			
3+	1274	407	0.78 (0.63,0.96)	0.0187	1.25 (0.91,1.71)	0.1665

**Note:** Both unadjusted and adjusted odds ratios shown in this table are estimated using logistic regression. Unadjusted odds ratio are based on separate logistic regressions for each predictor variable with that variable as the only predictor variable. Adjusted odds ratios are based on a single logistic regression consisting

of all the predictor variables in the table. For any given predictor variable in the adjusted column, the set of control variables consists of all the other predictor variables in the table

42 women reported to currently using contraceptives whereas they were pregnant/breastfeeding

^a^Contains 8 missing observations (but less than 10%)

### Reasons for discontinuing using contraceptives over the past two years

A total of 1054 women reported some use of contraceptives over the past 2 years, and reported their history of contraceptive use over the previous 2 years. Of these 1054 women, 383 (36%) reported 394 discontinuation events for contraceptive methods, and 66 switching events over the past 2 years (Table 5). There were 98 women who stopped because they desired to have a child, and among them 49 (50%) reported being pregnant prior to the interview date. The remaining 343 women who discontinued contraceptives, did not report wanting to get pregnant, although 129 (38%) of them reported a pregnancy following discontinuation (Table 5). The reasons for discontinuation included infrequent sex *n* = 45 (13.1%), inconvenient to use *n* = 19 (1.4%), health concerns *n* = 89 (20.2%) and other reasons *n* = 49 (11.1%). There were 122 women who reported no reason for discontinuing contraceptive use, of which 83 (68%) became pregnant within 3 months of discontinuation, and an additional six women reported contraceptive failure as the reason for the pregnancy. Over the 2-year period, there were a total of 89 pregnancies in the 1054 women who reported taking contraceptives prior to the pregnancy.

### Contraceptive discontinuation rates

There were 15 women who reported sterilisation as their contraceptive method, and who were excluded from the calculation of discontinuation rates for contraceptive use. A further 102 women only reported contraceptive use in the last 3 months prior to the survey and were also excluded from the discontinuation rates estimation, as women may be unaware of contraception failure if it happened in the 3 months prior to the survey. Table 4 shows the percentage of the 952 women who discontinued contraception, for all methods, and for each specific method, at 6 and 12 months. The overall discontinuation rates for the 952 women who reported using a contraceptive method during the 21 months were 15.8% at 6 months and 30.5% at 12 months. However, some of these women switched to another method during the study period, with 58 (6.1%) women using more than one method of contraception during the study period, sometimes immediately following the discontinuation and other times after a period of non-use (Table 5).

**Table 4:** Life table discontinuation rates for 1056 women in Kisesa in 2015/16, and median duration of use by method

Method	6-month	12-month	Median
	discontinuation	discontinuation	duration of
	rate	rate	use (in months)
Pills	27.8	43.6	15.5
Injections	21	41.9	15.5
Implants	12.8	24.9	–
IUD	9.6	13.7	–
All methods (Including traditional methods)	15.8	30.5	–
All modern methods (excluding sterilization)	16.9	32	21

**Table 5:** Reasons for discontinuation among women who used contraceptives in the past two years

	Number of	Discontinuation	Women who
	women	events	became pregnant (%)
Did not discontinue	613	0	0
Switched	58	66	0
Discontinued	383	394	178 (45.2)
Desired for pregnancy	98 (22.2)	100	49 (50)
Not reported desired for pregnancy	343 (77.8)		129 (38)
Reasons for discontinuation			
Infrequent sex	58 (13.1)		6 (10.3)
Health concerns	89 (20.2)		13 (14.6)
Inconvenient to use	19 (4.3)		4 (21.1)
Contraceptive failure	6 (1.4)		6 (100)
Other reasons	49 (11.1)		17 (34.7)
No reason reported	122 (27.7)		83 (68.0)

The median duration of use for all modern methods (except for sterilization) was 21 months. Pills and injections show a similar pattern of discontinuation with around 40% of women discontinuing within 12 months, and median use of 15.5 months. A lower discontinuation rate was estimated for women using intrauterine devices (IUD) or implants methods with only 9.6% of IUD users, and 12.8% of women who used implants stopping within 6 months. The median time for discontinuation of these methods was longer than the observation period of the study.

Results of the Cox regression on the factors associated with discontinuation in 952 women who used contraception during the past 2 years are shown on Table 6. All coefficients in the table, pass the assumption of proportional hazards. As expected, discontinuation varied by method and was significantly higher among users of the pills (aHR; 1.86, 95% CI 1.25,2.77) and injection (aHR; 2.04, 95% CI 1.59,2.77) as compared to users of implants. Further, results show that being over 25 years old, is significantly associated with a decreased rate of method discontinuation. Notwithstanding, HIV status, number of living children and education are not statistically associated with discontinuation of contraceptive use.

**Table 6:** Cox proportional hazards model on time until discontinuation of baseline method, by demographic. characteristics, number of living children, HIV status, and method type, *N* = 952.

Factors	Number of discontinuations	Rates (95% CI) per 100 women	Unadj HRs (95% CI)	*p*-value	Adjusted HRs (95% CI)	*p*-value
HIV status						
Negative	341	3.17 (2.85,3.52)	1			
Positive	32	2.94 (2.08,4.16)	0.83 (0.59,1.17)	0.2800	0.89 (0.63,1.29)	0.5200
Methods						
Implants	90	2.17 (1.76,2.66)	1			
Pills	35	3.70 (2.66,5.16)	1.73 (1.17,2.56)	0.0060	1.86 (1.25,2.77)	0.0020
Injection	225	4.19 (3.68,4.78)	1.97 (1.54,2.52)	0.0001	2.04 (1.59,2.61)	0.0001
IUD	11	1.24 (0.69,2.25)	0.56 (0.30,1.05)	0.0710	0.60 (0.32,1.13)	0.1160
Other methods	12	2.08 (1.18,3.66)	0.96 (0.52,1.75)	0.8850	1.07 (0.58,1.99)	0.8190
Age						
15–24	92	3.90 (3.18,4.78)	1			
25–34	184	3.05 (2.64,3.53)	0.77 (0.60,0.99)	0.0390	0.72 (0.56,0.93)	0.0110
35+	97	2.74 (2.25,3.35)	0.68 (0.51,0.90)	0.0080	0.67 (0.50,0.90)	0.0070
Education						
None/some primary	98	3.32 (2.72,4.05)	1			
Completed primary	222	3.06 (2.68,3.48)	0.92 (0.72,1.16)	0.4700		
Secondary+	52	3.05 (2.33,4.01)	0.92 (0.66,1.29)	0.6180	–	–
Number of living children						
*<* 3	127	3.44 (2.89,4.09)	1			
3+	246	2.99 (2.64,3.39)	0.86 (0.69,1.06)	0.160	–	–

## Discussion

Access to contraception is a crucial goal for sustainable development in many LMIC including Tanzania [18]. The policies on access to contraception are important for family planning, and also for prevention of mother to child transmission of HIV (PMTCT). Our results show that 16% of sexually active women in this rural area of Tanzania were currently using contraceptives, while 20% were currently pregnant or amenorrheic. Among all sexually experienced women of reproductive age (15–49 years), only 31% had ever used contraception, although 86% women in this population knew about family planning and contraceptive methods. The low use of contraception in this study concurs with previous studies showing low usage of modern contraceptives in other countries in sub-Saharan Africa [19, 20]. Injections, implants and pills were found to be the most common contraceptive methods used by women in this study. There were few women who used condoms (either male or female) for contraception, female sterilization, or who reported using traditional methods of contraception.

In this study 66% of all sexually-active, non-pregnant women who wanted to avoid pregnancy had an unmet need for contraception. We did not ask pregnant women about whether their pregnancy was wanted or not, and are therefore unable to ascertain the overall unmet need for contraception in this study. The unmet need for contraception in this study is higher than the unmet need for contraception reported in Uganda (34%) or Ghana (36%) [21].

Our results agree with the paper by MacQuarrie et al. [22] as we find no significant difference in the use of contraception by HIV status. However, women who were older, had attained higher educational levels and were married, had significantly higher levels of current contraceptive use. The positive association between education and contraceptive use is in line with findings from other studies [14, 23]. Women who have more years of education are more likely to understand the uses of contraception in reducing fertility, maternal and child morbidity and mortality. Educated women can also avoid the negative effects of family planning methods because they might have an awareness of the side effects of contraceptive methods, and be able to choose the most convenient method to use, and thereby increasing their consistent use [23]. In this study only 14% of the women had any secondary education, and the contraceptive discontinuation rate was lower in women with primary and secondary education compared to those with no education.

This study also showed around one third of women discontinue contraceptive use within a year, although this varies according to the type of contraceptives. The discontinuation rates were lowest among women using IUD (13.7% per year) or implants (24.9% per year), and higher for women using pills (43.6% per year) or injections (41.9% per year). The discontinuation rate for IUD is similar to that reported in Pakistan (19.1% in 10 months) and Senegal (18.4% in 12 months) [24, 25]. The discontinuation rates were lower among users of IUD and implants methods because these methods are long-lasting, and also removal of the devices requires help from a health professional [26]. Age, education and parity had a significant effect on contraceptive discontinuations, and discontinuation rates were higher among women who used pills and injections than those who used implants.

To understand the low contraceptive usage and high rates of discontinuation in this study, women were asked their reasons for stopping contraceptive use over the past 2 years. Around a quarter of the women who discontinued contraceptive use said they discontinued because they wanted to get pregnant. A further 28% become pregnant without giving a reason, perhaps reflecting an unplanned pregnancy, although only 6 women gave contraceptive failure as the reason for the pregnancy. Other than pregnancy, the most cited reasons for discontinuation of the use of contraceptive methods were fear of side effects, health concerns, inconvenience to use and infrequent sex. There was a very low discontinuation due to cost, problems in accessing contraception or marital dissolution. No one reported stopping due to religious or family opposition to contraceptive use.

This study shows that Tanzania is a long way from reaching its national family planning target of 60% contraceptive prevalence rate [27]. Results from this analysis emphasize the low uptake of contraception, and the high rates of contraceptive discontinuation, even among women who want to avoid pregnancy. Providing education on contraception, and universal access to contraceptives and family planning would empower women to overcome cultural barriers to the use of contraception. This would enable them to choose the number and spacing of children, to prevent unintended pregnancies, to reduce vertical transmission of HIV, and ultimately to improve maternal and child health. The high unmet need in this population underscores the importance of improving and expanding the provision of contraceptive use for women through different programmes, and making those programmes more accessible to younger women. Despite the benefits of contraception in preventing vertical transmission of HIV, the use of contraception was no higher among HIV positive women than HIV negative women. The PMTCT programme could be used to encourage HIV positive women to increase their use contraception for family planning and HIV prevention purposes.

### Strengths and limitations

This study reports on the behaviour of a large sample of sexually-active women of reproductive age, living in a defined area covered by the Kisesa HDSS. It reports the current use of contraception of both married and unmarried women. One limitation is that, it relies on the woman’s report of contraception use, and women may not accurately recall this over the 2 years. Secondly, young, unmarried women might not be honest regarding sexual debut, and they are the least likely to access contraception, or report contraception use, and this may underestimate the unmet need for contraception in this population. Thirdly, the study did not ask pregnant women or those with postpartum amenorrhea whether the pregnancy was intended or not, which may also lead to an underestimate of the unmet need for contraception. There are methodological limitations around censoring of episodes at the beginning and end of the 24-month calendar of contraceptive use, which may underestimate changes in contraceptive use, and hence the discontinuation rate. Finally, the study failed to take into consideration the competing risk events (such as switching between methods), and considered all switches to be discontinuation events, which would over-estimate the discontinuation rate in this population.

## Conclusions and implications

In conclusion, findings showed that a large proportion of sexually active women were not using any modern contraceptive, despite their high knowledge of contraceptives. Almost one fifth of sexually-active women had an unmet need for contraception in this population, which if translated across Tanzania would mean 2 million women needing contraceptive services. Using the Kisesa HDSS, we found non-significant association between women’s HIV status and both contraceptive use and discontinuation. Although there was no significant association, none of the HIV positive woman reported using condoms. There were significant relationships between contraceptive use and contraceptive discontinuation with age, education and parity. The predictors of contraceptive discontinuations indicated that, better interventions should be developed to target the needs of younger women. Women who used pills or injections had the highest rates of discontinuation, and a wider choice of long-acting contraceptive methods with better effectiveness and convenience may be better suited to their needs.

## Data Availability

The data from the sero-survey are openly available to collaborators. To obtain a copy of the data please write to Mark Urassa, PI TAZAMA study, PO Box 1462, Mwanza, Tanzania, requesting a copy of the data sharing agreement.

## Abbreviations

95% CI: 95% confidence interval
aHR: adjusted hazard ratios
CPR: Contraceptive prevalence rate
CTC: Care and Treatment Centre
HDSS: Health and Demographic Surveillance System
IUD: Intrauterine devices
LMIC: Low and middle income countries
LSHTM: London School of Hygiene & Tropical Medicine
LZIRB: Lake Zone Institutional Review Board
MMR: Maternal mortality ratio
OR: Odds Ratio
PMTCT: Prevention of mother-to-child transmission
SSA: Sub-Saharan Africa
TDHS: Tanzania Demographic and Health Survey
VCT: Voluntary Counselling and Testing
